# Physical Activity and Its Association with Depression in the Diabetic Hispanic Population

**DOI:** 10.7759/cureus.4981

**Published:** 2019-06-24

**Authors:** Sukaina Rizvi, Ali M Khan

**Affiliations:** 1 Psychiatry, Manhattan Psychiatric Center, Manhattan, USA; 2 Psychiatry, University of Texas Rio Grande Valley, Harlingen, USA

**Keywords:** phq-2, phq-9, hispanics, exercise, diabetes

## Abstract

Introduction

Hispanics are the largest ethnic minority group in the United States. The prevalence of depression and co-morbid depression in the Hispanic population is well-recognized. The positive association between physical activity and psychological health improves mood, emotional well-being, and prognostic outcome.

Objectives

There are two aspects of our research paper. First, it critically reviews the available literature showing the correlation between physical exercise and depression. Second, it analyzes the association between exercise and depression in uncontrolled diabetic Hispanics using data collected from the local community intervention program.

Method

A chi-square analysis was conducted to examine whether levels of physical activity reported at the baseline were associated with the frequency of depressed mood and anhedonia self-reported for the previous two weeks. This study utilized the use of the PHQ-2 scale for the assessment of depressive symptoms. The PHQ-2 scale is a useful tool to screen for depression in the integrated care setting. Participants from a local community intervention program were stratified on the basis of their gender and preferred language. Data were collected and represented in tables according to demographic characteristics.

Results

Our study established a statistically significant association between the levels of physical activity and the frequency of depression symptoms among Spanish speaking participants from the local community intervention program. These results provide convincing evidence that biological, developmental, social, and psychological factors facilitate the association between physical activity and depression.

## Introduction

Depression is a chronic debilitating mental condition that affects mood, leading to severe functional impairment in everyday life. Depression is bidirectionally associated with diabetes, which can further intensify morbidity, mortality, and disability. The prevalence rates of depression are significantly higher in the diabetic population. The co-morbidity of depression affecting adults with diabetes is about 15%-30% [1]. Research has speculated on the frequent co-occurrence of depression and diabetes in the Hispanic population than others [2]. Our study will be limited to the Hispanic population since cultural and racial factors can impact the occurrence of certain diseases and their co-morbid conditions. As per the literature review, physical activity has a promising potential in improving the depressive symptoms and clinical outcome in people suffering from depression. This article has been written to describe the effects of physical activity on depression in the diabetic Hispanic population in specific.

## Materials and methods

Methods

This study summarizes the results of analyses using data collected from the local community intervention program. To be eligible for the program, individuals must reside in the Rio Grande Valley, have uncontrolled diabetes (HBA1C>8.0), be over the age of 18 years, and be uninsured on Medicare/Medicaid. A chi-square analysis was conducted to examine whether the levels of physical activity reported at baseline were associated with the frequency of depressed mood and anhedonia self-reported for the previous two weeks.

Description of survey instruments

At baseline, participants were administered the short International Physical Activity Questionnaire (IPAQ) and the Patient Health Questionnaire-2 (PHQ-2). The PHQ-2 is a screening tool that includes the first two items from the PHQ-9 (major depressive disorders) that assess the frequency of depressed mood and anhedonia over the past two weeks. For these analyses, we limited data to participants (n=5740) for which PHQ-2 and physical activity responses were not missing (n=1,627). Given that 72% of observations were excluded due to missing data, a table was prepared to summarize the socio-demographic characteristics between included and excluded individuals (Table 1).

**Table 1:** Summary of demographic characteristics for individuals with missing data for Patient Health Questionnaire -2 (PHQ-2) and physical activity

	Missing Data on Physical Activity or Patient Health Questionnaire-2 (PHQ-2)		
	Yes (%)	No (%)	χ^2 ^p-value
Age (years)			
18-29	61 (2)	27 (2)	0.8633
30-39	311 (8)	121 (7)	
40-49	868 (21)	358 (22)	
50-59	1454 (35)	581 (36)	
60+	1417 (34)	540 (33)	
Gender			
Female	2822 (69)	1100 (68)	0.4618
Male	1291 (31)	527 (32)	
Preferred Language			
Spanish	2870 (70)	1056 (65)	0.0004
English	1230 (30)	565 (35)	
Education			
Less than High School	2366 (68)	955 (64)	0.0071
High School/GED	593 (17)	274 (18)	
Some college or greater	519 (15)	269 (18)	
Previous Diagnosis of Depression			
Yes	702 (20)	476 (30)	<0.0001
No	3035 (80)	1146 (70)	

The total metabolic equivalent (MET)-adjusted minutes of exercise per week was calculated by summing the total number of MET-adjusted minutes for mild, moderate, and vigorous exercise activities. Four levels of physical activity were defined: Sedentary (0 MET), Low (1-599 MET), Moderate (600-1499 MET), and High (≥1500 MET). An indicator variable for likely major depressive disorder (PHQ-2 score ≥3) was created by categorizing the PHQ-2 score (range 0-6). Demographic information collected included age, gender, preferred language (English/Spanish), education, and annual household income (CR1). Participants were also asked if they had ever been diagnosed with depression. We considered a previous diagnosis of depression as a potential confounder of the association between physical activity and depression symptoms. A chi-square test was used to examine the association between physical activity and the frequency of depression symptoms. We repeated the test stratified for individuals with and without a previous diagnosis of depression to examine the potential impact of confounding on the association of interest. We performed similar analyses for gender and preferred language. We used the Cochran-Mantel-Haenszel (CMH) chi-square test to determine whether the association remained the same after adjustment. All findings are presented in Tables 2-4

**Table 2:** Summary of demographic characteristics for participants from the community intervention program by the frequency of depressed mood and anhedonia over the past two weeks (n=1627)

	Major Depressive Disorder Likely		
	Yes (%)	No (%)	χ^2 ^p-value
Age (years)			
18-29	3 (1)	24 (2)	0.1932
30-39	17 (6)	104 (8)	
40-49	78 (25)	280 (21)	
50-59	116 (38)	465 (35)	
60+	94 (30)	446 (34)	
Gender			
Female	232 (75)	868 (66)	0.0013
Male	76 (25)	451 (34)	
Preferred Language			
Spanish	184 (60)	872 (66)	0.0334
English	123 (40)	442 (34)	
Education			
Less than High School	178 (66)	777 (63)	0.1141
High School/GED	55 (20)	219 (18)	
Some college or greater	37 (14)	232 (19)	
Previous Diagnosis of Depression			
Yes	202 (66)	274 (21)	<0.0001
No	103 (34)	1043 (79)	

**Table 3:** Levels of physical activity by frequency of depressed mood and anhedonia over the past two weeks, local community intervention program participants (n=1627)

	Major Depressive Disorder Likely		
	Yes n (%)	No n (%)	χ^2 ^p-value
Physical Activity (Total metabolic equivalent MET-adjusted minutes)			
Sedentary	255 (83)	1012 (77)	0.0150
Low	24 (8)	97 (7)	
Moderate	13 (4)	129 (10)	
High	16 (5)	81 (6)	

**Table 4:** Levels of physical activity by frequency of depressed mood and anhedonia over the past two weeks, local community intervention program participants stratified by gender and preferred language (n=1627)

	Major Depressive Disorder Likely		
	Yes (%)	No (%)	χ^2 ^p-value
No Previous Diagnosis of Depression			
Physical Activity (Total metabolic equivalent MET-adjusted minutes)			
Sedentary	85 (82)	798 (76)	0.1847
Low	8 (8)	68 (7)	
Moderate	4 (4)	109 (10)	
High	6 (6)	68 (7)	
Previous Diagnosis of Depression			
Physical Activity (Total metabolic equivalent MET-adjusted minutes)			
Sedentary	167 (83)	212 (77)	0.4175
Low	16 (8)	29 (11)	
Moderate	9 (4)	20 (7)	
High	10 (5)	13 (5)	
Females			
Physical Activity (Total metabolic equivalent MET-adjusted minutes)			
Sedentary	195 (84)	681 (78)	0.1125
Low	18 (8)	67 (8)	
Moderate	10 (4)	77 (9)	
High	9 (4)	43 (5)	
Males			
Physical Activity (Total metabolic equivalent MET-adjusted minutes)			
Sedentary	60 (79)	331 (73)	0.2564
Low	6 (8)	30 (7)	
Moderate	3 (4)	52 (12)	
High	7(9)	38 (8)	
English Preferred			
Physical Activity (Total metabolic equivalent MET-adjusted minutes)			
Sedentary	96 (78)	323 (73)	0.5557
Low	11 (8)	40 (9)	
Moderate	8 (7)	47 (11)	
High	8 (7)	32 (7)	
Spanish Preferred			
Physical Activity (Total metabolic equivalent MET-adjusted minutes)			
Sedentary	159 (86)	684 (78)	0.0193
Low	12 (7)	57 (7)	
Moderate	5 (3)	82 (9)	
High	8 (4)	49 (6)	

## Results

Participants with missing data were more likely to be Spanish speakers, to have less than high school education, and approximately 10% less likely to have self-reported the diagnosis of depression at baseline (Table 1). Local community intervention program participants that experienced depressed mood and anhedonia over the past two weeks were more likely to be women, preferred English speakers, and significantly more likely to have self-reported a previous diagnosis of depression (66% vs 21%) when compared to participants for whom major depressive disorder was found to be unlikely (Table 2).

A statistically significant association was observed between levels of physical activity and frequency of depression symptoms among participants (p-value 0.0150). Individuals experiencing depressive symptoms were more likely to report a sedentary level of physical activity (83%) when compared to those not experiencing depressive symptoms (77%). Confounding of the association by a previous diagnosis of depression did not occur, but the results suggest that the association between physical activity and depressive symptoms differed by gender and language (CMH p-value<0.05) (Table 3 and Table 4). Overall, the association between physical activity and depressive symptoms only remained statistically significant among Spanish speakers. Results should be interpreted cautiously given the potential for selection bias and confounding.

## Discussion

The term Hispanic broadly refers to the group of people who have some historical connection to the Spanish language. The U.S Census Bureau defines the term Hispanic or Latino as a person of Cuba Mexico, Puerto Rica, South or Central American, or other Spanish culture origin regardless of race.

Depression is one of the leading causes of disability in the United States and millions have been spent to treat this disorder [3-4]. It is suspected that, by the year 2020, at least 17% of Americans will suffer from a major depressive episode at least once in their life [5]. The rate of depression has varied greatly from population to population. The low socio-economic population with chronic illnesses have a higher risk of co-morbid depression as compared to the general population, with the rate in Hispanics as high as 33% [2,6]. Hispanics describe depressive symptoms as a lack of motivation to participate in family matters, apathy, and sadness. Studies of Hispanic culture revealed multiple factors that strongly correlate to the overall rate of depression in the Hispanic population. Exposure to environmental and psychosocial burdens, such as low economic status, religion, gender roles, family customs, health beliefs, single parenting, immigration issues, acculturation, and enculturation in American society, attributes to the depressive disorder. Liang et al. speculated higher levels of depressive symptoms in middle-aged Hispanics and blacks than in the white American middle-aged population [1,7].

Now the question arises, how do you know or measure the severity of depression? To measure the severity of depression, a questionnaire called the Patient Health Questionnaire 2, better known as the PHQ-2, is used. PHQ2 is a preliminary screening tool in integrated care settings, which is administered prior to performing PHQ-9. PHQ-2 is the first step in identifying depressive disorders. The individual is asked two simple questions about mood and anhedonia over the past two weeks. The answers are given a score out of 3 for each question. The points of both the answers are added. If the patient answers no to each question, then no further intervention is required. If the PHQ-2 score is 3 or more, then further screening is required by Patient Health Questionnaire 9 or PHQ-9. PHQ-9 is used to confirm positive PHQ-2 results. It is used to determine if the patient meets the criteria for a depressive disorder. PHQ-9 has nine questions, with each question carrying three points. The entire questionnaire totals 27 points out of which 15 are the benchmark that a patient must achieve before starting an intervention. PHQ2 and PHQ9 are publicly available free-of-cost tools.

A study was conducted to validate PHQ-2 and PHQ-9. Consecutive adult patients attending Auckland family practices completed the PHQ-9, which was followed by the completion of a Composite International Diagnostic Interview (CIDI) depression reference standard. The sensitivities and specificities for both PHQ-2 and PHQ-9 were analyzed. In a sample population of 2642 patients who completed both the PHQ 9 and CIDI, for PHQ-2 scores of 2 or higher, the sensitivity and specificity were 86% and 78%, respectively, and for a PHQ-2 score of 3 or higher, the sensitivity and specificity were 61% and 92%. For a PHQ 9 score of 10 or higher, the results were 74% and 91%. After conducting this analysis, it was noticed that a PHQ-2 score of 2 or higher detected more cases of depression compared to a PHQ 2 score of 3 or more. For PHQ-9, a score of 10 or higher detected more cases as compared to the PHQ determination of major depression originally described by Spitzer et al. in 1999 [8]. The participants were split into three groups, and each person was given a random questionnaire, the PHQ 9, the two Questions with Help questionnaire, or the CIDI, which was used as a control measure. The questionnaires were filled in separate rooms. The data was gathered and analyzed using the Centre for Evidence based medicine calculator on the University of Toronto website (http://www.cebm.utoronto.ca). The results were then generated. In another study conducted in the Latvian and Russian languages using PHQ 2 and PHQ 9 and using the Mini International Neuropsychiatric Interview (MINI) as a control in Latvia. There were 1467 patients who completed PHQ 9 and MINI. Overall, the PHQ-9 items showed good internal reliability (Cronbach’s alpha 0.81 for the Latvian version and 0.79 for the Russian version of the PHQ-9). A cut-off score of 8 or greater was established for PHQ-9 (sensitivity 0.75 and 0.79, specificity 0.84 and 0.80 for the Latvian and Russian languages, respectively). For PHQ-2, a score of 2 or higher (sensitivity 0.79 and 0.79, specificity 0.65 and 0.67 for the Latvian and Russian languages) detected more cases of depression than a score of 3 or higher [9].

PHQ-2 asks two simple questions and its efficacy is being compared to longer screening tools, such as the Beck Depression Inventory or the Zhung Depression scale [10-12]. Kroenke et al. speculated the validity of the two-item depression scale in 6000 patients in primary care settings. They demonstrated an inverse correlation between PHQ-2 severity and patient’s functionality in everyday life [13]. Chunyu Li et al. postulated the validity of PHQ-2 as a screening tool for depression in older people when they found an increase in the specificity of test with age [14]. However, Richardson LP concluded promising results in the sensitivity and specificity of PHQ-2 for detecting depression in adolescent individuals in the outpatient setting [15].

Depression is recognized as a potential risk factor for the onset of other medical comorbidities, including diabetes. Recent studies have shown that depression impacts the mortality and morbidity of diabetes by affecting treatment compliance and self-management particularly in the older population [16]. Depression and diabetes co-occur more frequently in the Hispanic population than in others. Hispanics presumed a strong correlation between emotional dysregulation and diabetes as part of their culturally bound belief. It is estimated that 25%-30% of older Mexican Americans have type-2 diabetes and 25% of these diabetic individuals report depressive symptoms. As per research data from the Hispanic Established Population for the Epidemiologic Study of the Elderly (EPESE), depression symptoms were associated with a poor prognostic outcome in diabetic Mexican Americans of older age in terms of disability and increased cost of health care [16-17]. This is consistent with findings from other studies, which also showed significantly higher HBA1C levels in depressed Latino participants as opposed to participants without depression [18].

 It is a common notion that physical activity and exercise have a positive impact on the physical health, mood, anxiety, and overall psychological well-being of an individual. The beneficial effects of exercise and physical activity are comparable to the effects of anxiolytics and antidepressants in healthy and depressed individuals [19-20]. This compels toward the idea that a lack of physical activity contributes to the onset of mental health disorders. Multiple epidemiological studies corroborated the inverse association between physical activity and depressive symptoms [21-23]. Earlier, Farmer et al. speculated decreased risk of depression with regular exercise in a study of 1,900 subjects [24]. The protective effect of exercise on mental health was also supported by Motl et al. [25]. Lack of physical activity not only increases the risk of depression but also attributes to diabetes. A randomized controlled trial was conducted to assess for mental health in 58 Puerto Rican adults with type-2 diabetes for 16 weeks. The trial utilized the use of high-intensity progressive resistance exercise training (PRT). They revealed that the incorporation of exercise training into the treatment plan resulted in a significant improvement in mental health on the Geriatric Depression scale [26]. Penninx et al. hypothesized the antidepressant role of aerobic exercise while Singh et al. emphasized resistance exercise as a possible antidepressant [27-28].

The exact cascade of events that lead to the beneficial effect of physical activity as an antidepressant is not well- recognized. However, research studies have attributed to developmental, psychological, and neurobiological factors to account for its efficacy as an antidepressant. Physical activity may lead to higher resilience, which serves as a protecting shield against mental trauma. Earlier studies have suggested increased central norepinephrine, serotonin synthesis, atrial natriuretic peptide, and beta-endorphins serve as biological mediators for a possible therapeutic effect in depression [23,29-30].

## Conclusions

Our research has analyzed the impact of physical activity on depression in the Hispanic diabetic population. The results of the study imply a convincing effect of physical activity on depression as validated by the statistically significant association between physical activity and depressive mood symptoms among Spanish speakers. Given the literature review and the above results, participation in physical activity is strongly associated with optimistic mood, low negativity, enhanced self-esteem, greater emotional, and social, psychological, and physical well-being. Therefore, the present study provides a well-established link to support physical activity as a potential antidepressant in the Hispanic population that may reduce the prevalence of depressive disorders, which ultimately decreases mortality, morbidity, and disability. For instance, the major limitations to our study were the inclusion of subjects who resided in the local community program. This study also establishes the need for future research in order to elucidate the role of high activity exercises in improving depression symptoms. Moreover, research is also needed to explore the possible role of sex, race, and spoken language differences, as they impact physical activity in the depressed patient population.
